# Postoperative and postdischarge nausea and vomiting following ambulatory eye, head, and neck surgeries: a retrospective cohort study comparing incidence and associated factors

**DOI:** 10.1186/s13741-024-00360-4

**Published:** 2024-01-20

**Authors:** Mark Xiao, Dongdong Yao, Kara G. Fields, Pankaj Sarin, Alvaro Andres Macias, Sunil Eappen, Jeremy Juang

**Affiliations:** 1 Department of Anesthesiology, Massachusetts Eye and Ear (MEE), 243 Charles St., Boston, MA 02114 USA; 2 Harvard Medical School, Boston, MA USA; 3 Department of Anesthesiology, Brigham and Women’s Hospital (BWH), 75 Francis St., Boston, MA 02115 USA; 4 Department of Anesthesia and Perioperative Care, University of California, San Francisco, CA USA

**Keywords:** Nausea, Vomiting, PONV, PDNV, Perioperative opioid usage, Ophthalmology, Otolaryngology, Ambulatory surgery, Anesthesia

## Abstract

**Background:**

Ambulatory surgery is often followed by the development of nausea and/or vomiting (N/V). Although risk factors for postoperative nausea and vomiting (PONV) are frequently discussed, the distinction between PONV and postdischarge nausea and vomiting (PDNV) is unclear. This is especially troublesome given the potential consequences of postdischarge nausea and vomiting (PDNV), which include major discomfort and hospital readmission.

**Methods:**

In this retrospective cohort study, data from 10,231 adult patients undergoing ambulatory ophthalmology or otolaryngology procedures with general anesthesia were collected and analyzed. Binary and multinomial logistic regression was used to assess the association between patient and anesthetic characteristics (including age, body mass index (BMI), American Society of Anesthesiologists Physical Status (ASA P/S) classification, current smoker status, and intra- and postoperative opioid usage) and the odds ratios of experiencing only PDNV, only PONV, or both PONV and PDNV, as compared to not experiencing N/V at all.

**Results:**

We found that 17.8% of all patients developed N/V (PONV and/or PDNV). Patients who experienced PONV had a 2.79 (95% confidence interval 2.24–3.46) times greater risk of reporting PDNV. Binary logistic regression found that younger age, opioid use, and female sex were associated with an increased likelihood of experiencing any N/V. Increased use of nitrous oxide and a higher ASA P/S class was associated with elevated likelihood of PONV, but not PDNV or PONV plus PDNV.

**Conclusions:**

Patients experiencing N/V in the PACU are observed to develop PDNV disproportionately by a factor of 2.79. The patients have distinct predictors, indicating important opportunities for care improvements beyond current guidelines.

**Supplementary Information:**

The online version contains supplementary material available at 10.1186/s13741-024-00360-4.

## Background

Postoperative nausea and vomiting (PONV) is one of the most frequent complications following ambulatory surgery, with an incidence rate upwards of 80% in certain high-risk patient groups and an overall rate ranging from 20 to 30% (Apfel et al. 1999; Cohen et al. 1994; Amirshahi et al. 2020; Oderda et al. 2019). Despite being commonplace, a previous survey revealed that patients are more averse to PONV than pain and other highly undesirable postoperative complications (Macario et al. 1999). Additionally, even moderate instances of PONV can lead to significant consequences, including delayed discharges, disrupted postanesthesia care unit (PACU) workflow, increased medical expenses, and diminished patient satisfaction (Gan 2002; Hill et al. 2000; Gress et al. 2020). In the context of ophthalmology and otolaryngology procedures, sequelae can also include increased intraocular pressure and disrupted suture lines, including wound contamination by acidic contents.

PONV occurrence in hospitals has been widely investigated and many risk factors of legacy status have been identified, including female sex, history of PONV, nonsmoker status, and intraoperative usage of opioid analgesics, among others (Apfel et al. 2012; Eberhart et al. 2000; Dziadzko and Aubrun 2020; Gan et al. 2020). However, recent evidence suggests that incidence and costs associated with PONV are higher than previously estimated, with rates ranging from 44 to 72% in inpatient procedures that utilize IV opioids (Oderda et al. 2019). Certain service types, including otolaryngology, ophthalmology, and gastroenterology, may be disproportionately impacted by PONV (Oderda et al. 2019; Gan et al. 2020; Sinclair David et al. 1999).

While PONV generally refers to nausea and vomiting (N/V) in the PACU, postdischarge nausea and vomiting (PDNV) occurs at home. While many sources define PONV as N/V within 24 h of a procedure (Amirshahi et al. 2020); for clarity purposes, and given the importance of locality, we delineate N/V based on occurrence before (PONV) or after (PDNV) discharge. The difference in environment, notably the distance from medical staff and services, may have consequential effects on patient well-being. Patients experiencing PDNV have few means to alleviate their discomfort and are observed to be disproportionately involved in hospital readmissions (Celio et al. 2019; Lerman 2019; Merna et al. 2019). As such, the prediction and mitigation of such postdischarge complications are of great interest. This is especially relevant for ambulatory surgeries as patients spend shorter durations in the PACU and less time under skilled nurse supervision before being sent home. Additionally, while PONV risk factors are well investigated, it is unknown if they similarly apply to patients who experience both PONV and PDNV. An updated analysis of new, robust data that differentiates PDNV from PONV is necessary to further inform clinical practice.

In this retrospective cohort study, we seek to compare the risk factors for and incidence of patients experiencing PONV and PDNV. We further characterize and compare patients by four groups: those who experience no N/V, PONV, PDNV, or both PONV and PDNV (Supplementary Tables S1 and S2, Additional file 1). A total of 10,231 patients who underwent ambulatory surgery with general anesthesia for ophthalmology or otolaryngology services were assessed for N/V in the PACU and reassessed at home via telephone on postoperative day one (POD1). Previous literature has found an increased frequency of PONV following ophthalmic and otolaryngologic procedures (Chung and Mezei 1999). Given their salience as potentially high-risk services, we focus our analysis on these two types of procedures. Our goals were to reevaluate whether previously identified risk factors for PONV also apply to PDNV, and secondly to identify whether less explored patient or anesthetic characteristics such as ASA class and surgery type are linked to the time and setting of N/V occurrence.

## Methods

The study was conducted at Massachusetts Eye and Ear (MEE) with MEE Institutional Review Board (IRB) approval (1199654–1/(18-026H)) and Massachusetts General Brigham IRB approval (Protocol # 2019P00194). The study was conducted in accordance with all rules and regulations laid out by the IRB and human studies committee. This manuscript adheres to applicable STROBE guidelines. A waiver of written informed consent was obtained for this study. Electronic medical records (EMR) of procedures performed by the otolaryngology and ophthalmology services between April 4, 2016, and May 4, 2020, were analyzed. Initial inclusion criteria were patients age 18 years or older, ambulatory surgery procedures, services provided by otolaryngology and ophthalmology, and general anesthesia as the primary anesthetic type. PACU N/V status was positive if nursing observed, or patient-reported, nausea or emesis, or if additional antiemetics or medications often used in response to N/V were administered in the PACU (ondansetron, metoclopramide, famotidine, dexamethasone, promethazine, and/or scopolamine patch antiemetics). PDNV status was positive if patients reported nausea or emesis during their POD1 follow-up phone call. Data regarding antiemetic use at home was not available nor analyzed. Cases with incomplete records were excluded. Each patient’s first procedure was included (all following procedures were excluded) to ensure unique patient-anesthetic encounters, with no patient-specific duplication. A resulting 10,231 procedures were identified and included in our analyses.

### Statistical analysis

Our primary investigation consisted of two comparisons: (i) PONV vs no PONV and (ii) PDNV vs no PDNV. Our secondary outcomes consisted of four categories: (i) no N/V, (ii) PONV, (iii) PDNV (N/V only at home), and (iv) PONV and PDNV. An a priori determined list of potential factors associated with the odds ratio of PONV and/or at PDNV included age, sex, body mass index (BMI), American Society of Anesthesiologists (ASA) class, current smoker status, prophylactic antiemetic administration (including ondansetron, dexamethasone, promethazine, or scopolamine patch antiemetics administered before the PACU), otolaryngology vs. ophthalmology procedure type, scopolamine patch, inhalational anesthesia duration, nitrous oxide duration, intraoperative total anesthesia duration, propofol only general anesthesia, PACU Phase 1 duration, and PACU Phase 2 duration. Cumulative hydromorphone, fentanyl, (oral) oxycodone, and morphine consumption were also used to calculate Milligram Morphine Equivalents (MME). The adjusted association of each factor on the a priori-defined list with the odds of being in each of the three N/V positive groups versus the No N/V group was assessed using multivariate logistic regression. Only the first surgery on each patient was included in the final analysis. Statistical analyses were performed using SAS software version 9.4 (SAS Institute, Cary, NC, USA).

### Sample size justification

The goal of building a multivariate logistic regression for PONV status was to identify predictors, not develop a prognostic model. However, De Jong et al. recommend at least 10 patients in the smallest outcome category per model parameter estimated in large sample to obtain adequate prediction model performance (Jong et al. 2019). A minimum of 200 patients in the smallest outcome category would be adequate to test the total of 20 parameters of interest identified a priori.

## Results

**Fig. 1:**
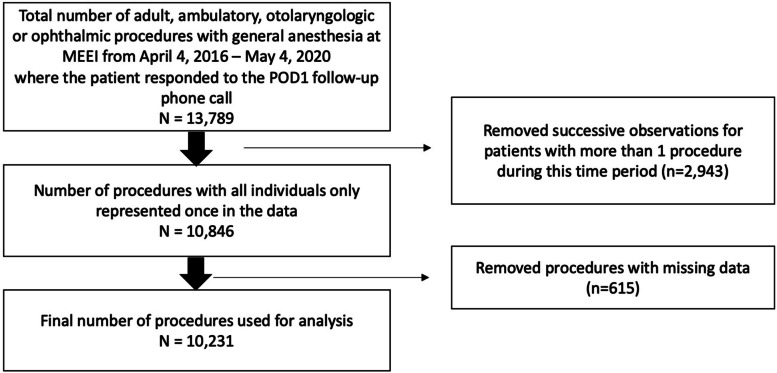
Flow diagram for study inclusion

Figure 1 depicts a flow diagram for study inclusion. Information from a total of 13,789 adult, ambulatory, otolaryngologic, or ophthalmic surgeries with general anesthesia was collected through POD1. To eliminate any correlation caused by patients who underwent multiple procedures, our analysis only included data from each patient’s first procedure. Observations with missing information were excluded. Data from a total of 10,231 unique patients were analyzed.

Table 1 depicts summary statistics for no PONV, PONV, no PDNV, and PDNV outcome groups for the explanatory factors considered. It contains independent information per outcome, including mean, standard deviation, count, and percentage when relevant. Table 2 reports the adjusted odds ratio (AOR) and 95% confidence intervals for the PONV vs no PONV outcome groups in comparison with the no N/V group, using the variables described in Table 1, as derived by binary logistic regression. Anesthesia characteristics were measured prior to the PACU. Table 3 reports AORs for the PDNV vs no PDNV groups. Table 3 includes PACU-specific variables, as well as previously used variables that were modified to include PACU data. Table 4 depicts the overall number and percentage of patients that fall into each outcome group.

**Table 1:** Summary statistics of no PONV, PONV, no PDNV, and PDNV

	No PONV (*n* = 8732)	PONV (*n* = 1499)	No PDNV (*n* = 9748)	PDNV (*n* = 483)
Patient characteristics				
Age (years)	51.4 ± 17.8	47.6 ± 16.2	51 ± 17.6	47 ± 17
Male Sex	4383 (50.2)	499 (33.3)	4714 (48.4)	168 (34.8)
ASA				
1	1440 (16.5)	326 (21.7)	1662 (17)	104 (21.5)
2	6038 (69.1)	1014 (67.6)	6727 (69)	325 (67.3)
3	1254 (14.4)	159 (10.6)	1359 (13.9)	54 (11.2)
Body mass index (kg/m^2^)	27.2 ± 5.6	27.2 ± 5.8	27.2 ± 5.6	26.5 ± 5.4
Current smoker	812 (9.3)	99 (6.6)	880 (9)	31 (6.4)
Service				
Otolaryngology	6933 (79.4)	1311 (87.5)	7837 (80.4)	407 (84.3)
Opthalmology	1799 (20.6)	188 (12.5)	1911 (19.6)	76 (15.7)
Pre/intraoperative anesthesia characteristics				
Total anesthesia time (minutes)	117.7 ± 61.4	135.2 ± 71.2	120.3 ± 63	119.8 ± 67.3
Inhalational time (minutes)	57 ± 60	88.6 ± 75.7	61.3 ± 63.1	69.2 ± 71.2
Nitrous time (minutes)	6.8 ± 22.7	12.6 ± 34.1	7.6 ± 24.7	8.4 ± 27.4
Propofol-only TIVA	1319 (15.1)	88 (5.9)	1374 (14.1)	33 (6.8)
Opioid use (MME)	20.8 ± 19.2	24.8 ± 19.7	21.2 ± 19.3	24.6 ± 19
Any opioid use	6409 (73.4)	1257 (83.9)	7260 (74.5)	406 (84.1)
Number of prophylactic antiemetics used				
0	188 (2.2)	24 (1.6)	204 (2.1)	8 (1.7)
1	1277 (14.6)	214 (14.3)	1430 (14.7)	61 (12.6)
2	6454 (73.9)	1021 (68.1)	7115 (73)	360 (74.5)
3	813 (9.3)	240 (16)	999 (10.2)	54 (11.2)
Pre/intraoperative/PACU anesthesia characteristics				
Opioid use (MME)			33.9 ± 26.4	42 ± 26
Any opioid use			8500 (87.2)	455 (94.2)
Number of prophylactic antiemetics				
0			170 (1.7)	6 (1.2)
1			1322 (13.6)	53 (11)
2			6994 (71.7)	346 (71.6)
3			1182 (12.1)	75 (15.5)
4			80 (0.8)	3 (0.6)
PACU phase 1 time (minutes)			66 ± 38.1	71.2 ± 30
PACU phase 2 time (minutes)			78.4 ± 49.8	97.2 ± 74.9
PONV			1333 (13.7)	166 (34.4)

Variables in the “Pre/intraoperative anesthesia characteristics” category refer to antiemetic and opioid drug use prior to the PACU while “Pre/intraoperative/PACU Anesthesia Characteristics” variables include all data collected in the PACU. The number of prophylactic antiemetics used variable in the “Pre/intraoperative anesthesia characteristics” category does not include promethazine due to the limited number of patients who received it pre-PACU (*n* = 4). The other antiemetics used include ondansetron, dexamethason, and scopolamine

*Abbreviations*: *N/V* nausea/vomiting, *PONV* postoperative nausea/vomiting, *AOR* adjusted odds ratio, *ASA* American Society of Anesthesiologists, *TIVA* total intravenous anesthesia, *MME* morphine milligram equivalents

**Table 2:** Binary logistic regression for PONV vs no PONV

Comparison	Outcome: PONV vs. no PONV	
	AOR (95% CI)	*p* value
Patient characteristics		
Age (per 1 year increase)	0.99 (0.99, 0.99)	< 0.001
Male sex	0.44 (0.39, 0.5)	< 0.001
ASA		
2 vs 1	0.81 (0.69, 0.94)	0.007
3 vs 1	0.77 (0.61, 0.97)	0.029
3 vs 2	0.95 (0.79, 1.15)	0.606
Body mass index (per 1 kg/m^2^ increase)	1.01 (1, 1.02)	0.155
Current smoker	0.7 (0.56, 0.87)	0.002
Otolaryngology service (vs. ophthalmology)	1.24 (1.04, 1.47)	0.015
Pre/intraoperative anesthesia characteristics		
Total anesthesia time (per 1 min increase)	1 (1, 1)	0.816
Inhalational time (per 1 min increase)	1.01 (1.01, 1.01)	< 0.001
Nitrous time (per 1 min increase)	1.01 (1, 1.01)	< 0.001
Propofol-only TIVA	0.69 (0.53, 0.91)	0.008
Number of prophylactic antiemetics		
1 vs. 0	1.09 (0.69, 1.73)	0.715
2 vs. 0	0.86 (0.55, 1.34)	0.503
3 vs. 0	1.17 (0.73, 1.86)	0.51
Opioid use (per 1 MME increase)	1 (1, 1.01)	0.042

Variables related to antiemetic and opioid drug use include all drugs administered pre-PACU. The number of prophylactic antiemetics used a variable (ondansetron, scopolamine, and dexamethasone) does not include promethazine due to the limited number of patients who received it pre-PACU (*n* = 4)

*Abbreviations*: *N/V* nausea/vomiting, *PONV* postoperative nausea/vomiting, *AOR* adjusted odds ratio, *ASA* American Society of Anesthesiologists, *TIVA* total intravenous anesthesia, *MME* morphine milligram equivalents

**Table 3:** Binary logistic regression for PDNV vs no PDNV

Comparison	Outcome: PDNV vs. no PDNV	
	AOR (95% CI)	*p* value
Patient characteristics		
Age (per 1 year increase)	0.99 (0.98, 1)	0.002
Male sex	0.63 (0.51, 0.76)	< 0.001
ASA		
2 vs 1	0.99 (0.77, 1.27)	0.945
3 vs 1	1.02 (0.69, 1.49)	0.935
3 vs 2	1.02 (0.75, 1.4)	0.876
Body mass index (per 1 kg/m^2^ increase)	0.98 (0.96, 0.99)	0.012
Current smoker	0.74 (0.51, 1.08)	0.115
Otolaryngology service ( vs. ophthalmology)	0.87 (0.66, 1.14)	0.319
Pre/intraoperative anesthesia characteristics		
Total anesthesia time (per 1 min increase)	1 (1, 1)	0.276
Inhalational time (per 1 min increase)	1 (1, 1)	0.77
Nitrous time (per 1 min increase)	1 (1, 1)	0.839
Propofol-only TIVA	0.62 (0.41, 0.94)	0.025
Pre/intraoperative/PACU anesthesia characteristics		
PACU phase 1 time (per 1 min increase)	1 (1, 1)	0.938
PACU phase 2 time (per 1 min increase)	1 (1, 1)	< 0.001
Number of antiemetics used		
1 vs. 0	0.84 (0.35, 1.98)	0.683
2 vs. 0	0.88 (0.38, 2.01)	0.757
3 vs. 0	0.67 (0.28, 1.59)	0.363
4 vs. 0	0.2 (0.05, 0.84)	0.028
Opioid use (per 1 MME increase)	1.01 (1, 1.01)	< 0.001
PONV	2.79 (2.24, 3.46)	< 0.001

Variables related to antiemetic and opioid drug use include all drugs administered intraoperatively and in the PACU. The antiemetics used variable includes ondanestron, scopolamine, dexamethasone, and promethazine *Abbreviations*: *N/V* nausea/vomiting, *PONV* postoperative nausea/vomiting, *PDNV* postdischarge nausea/vomiting, *AOR* adjusted odds ratio, *ASA* American Society of Anesthesiologists, *TIVA* total intravenous anesthesia, *MME* morphine milligram equivalents

**Table 4:** Incidence of all outcome groups

	PDNV	No PDNV	Row totals
**PONV**	166	1333	1499 (14%)
**No PONV**	317	8415	8732 (86%)
**Column totals**	483 (4%)	9748 (96%)	10,231

*Abbreviations*: *PONV* postoperative nausea and vomiting, *PDNV* postdischarge nausea and vomiting

Higher age, female sex, and opioid use were significant high-risk indicators (*p* < 0.05) for both PONV and PDNV. As expected, propofol-only TIVA was similarly associated with decreased incidence for both groups. BMI was associated with a decreased risk of PDNV. Smoking status and higher ASA class were associated with decreased odds of developing PONV, but not PDNV. Longer durations of inhalational anesthesia and nitrous oxide use were associated with an increased risk of PONV by 1% for each additional minute. Otolaryngologic procedures were associated with a greater risk of PONV. PACU phase 2 time and PONV were also factors associated with PDNV.

N/V occurred in a total of 1816 patients (17.8%). One hundred and sixty-six patients (11.1%) who experienced PONV also reported PDNV. In contrast, only 317 (3.6%) of patients who did not have PONV later developed PDNV (Table 4). Patients who experienced PONV had 2.79 times the risk of reporting PDNV than those who did not.

## Discussion

Due to its prevalence and its associated costs, the prevention and management of PONV is highly investigated. However, there is a comparative lack of literature that directly compares the incidence of and risk factors for PONV, PDNV, and both PONV and PDNV. Our study finds that a distinction does exist between these outcomes and further attention is warranted.

In accordance with the 20–30% incidence rate reported by previous literature (Apfel et al. 1999; Cohen et al. 1994; Amirshahi et al. 2020), we found that 17.8% of patients experienced N/V following ambulatory ophthalmologic or otolaryngologic surgery with general anesthesia in at least one setting and that 11.1% of patients experiencing PONV will go on to develop PDNV. Also corresponding with previous literature, female sex, and younger age were observed to be associated with PONV and PDNV (Apfel et al. 2012). However, we did find salient differences in associated factors. In particular, the use of volatile anesthetics and nitrous oxide, known as positive risk factors for N/V (Gan et al. 2020), were only significant in the PACU. This may be due to the transient nature of volatile anesthetics, as any remnant physiological effects are unlikely to be salient on POD1. Our results also authenticate the previous finding that propofol-only TIVA was predictive of decreased PONV and PDNV (Williams et al. 2023).

Our findings suggest that a lower ASA/PS status and otolaryngologic service type, more controversial risk factors (Gan et al. 2020), were associated with PONV but not PDNV. Together, this suggests that while patient characteristics influence the occurrence of PONV, PDNV is primarily driven by longer-lasting anesthesia-mediated effects. PACU phase 2 durations were associated with PDNV even when controlling for all other factors. Greater postdischarge attention should be paid to patients who take longer to get discharged by the PACU, whether it be due to N/V or any other reason.

A previous article by Williams et al. revealed that five-drug prophylaxis was associated with a 0.15 odds ratio for developing PONV (Williams et al. 2023). We further corroborate this finding as patients who received four total antiemetics (the maximum in our study) had a similarly low risk of PDNV (0.2 AOR). This finding emphasizes the prophylactic role of promethazine in particular, as it was primarily administered in the PACU (as opposed to the other antiemetics used).

Estimates of PDNV following ambulatory surgery vary, ranging from 14 to 60% (Apfel et al. 2012; Efune et al. 2018; Odom-Forren et al. 2013). We estimate that patients who experienced N/V in the PACU have a 2.79 times greater risk of developing PDNV. The overall incidence of PDNV in our study was small (4.7%), but our findings were consistent with previous findings that range from 2.78 to 3.14-fold increases in risk (Apfel et al. 2012; Williams et al. 2023; Odom-Forren et al. 2013). One potential reason for our low PDNV rate may be due to differences in sampling: while other studies with higher rates of PDNV assessed patients for up to a week postsurgery, this study only assessed PDNV on POD1 (Apfel et al. 2012; Odom-Forren et al. 2013).

As an observational study, selection bias is an inherent limitation. For instance, we found that the number of prophylactic antiemetics used was not associated with decreased incidence of PONV. Patients at higher risk of PONV may be more likely to receive antiemetic prophylaxis, which may lead to type 2 errors. Additionally, a recent network meta-analysis Cochrane review highlighted that scopolamine in combination with aprepitant or antidopaminergic may hinder their individual anti-vomiting effects (Weibel et al. 2020). In our study, such combinations were not common intraoperatively. While we did not find any “cancellation” effects between scopolamine and promethazine (Supplementary Table S3, Additional file 1) in the incidence of PDNV, anesthesia providers should be cognizant of the potential interactions when using antiemetics in combination, particularly in the PACU when drug concentrations in vivo are highest.

## Conclusion

In conclusion, the population of patients who develop PDNV or both PONV and PDNV following ambulatory ophthalmic/otolaryngologic surgery are important targets for care improvements beyond those predicated on legacy guidelines. We observe that these patients have associated factors that are distinct from currently known PONV risk factors.

### Supplementary Information

**Additional file 1: Supplementary Table 1.** Summary Statistics: Patient and Operative Factors by N/V Status. **Supplementary Table 2.** Multivariable Adjusted Odds Ratios. **Supplementary Table 3.** Binary Logistic Regression for PDNV vs No PDNV.

## Data Availability

The datasets used and analyzed during the current study are available from the corresponding author on reasonable request.

## Abbreviations

N/V: Nausea and vomiting
PONV: Postoperative nausea and vomiting (occurring in the PACU)
PDNV: Postdischarge nausea and vomiting (occurring at home)
No N/V: N/V neither reported in the PACU nor at home
BMI: Body mass index
ASA: American Society of Anesthesiologists
PACU: Postanesthesia care unit
POD1: Postoperative day 1
MEE: Massachusetts Eye and Ear
IRB: Institutional Review Board
EMR: Electronic Medical Records
AOR: Adjusted odds ratio
